# Quantum halo states in two-dimensional dipolar clusters

**DOI:** 10.1038/s41598-021-98838-4

**Published:** 2021-09-30

**Authors:** G. Guijarro, G. E. Astrakharchik, J. Boronat

**Affiliations:** Departament de Física, Campus Nord B4-B5, Universitat Politècnica de Catalunya, 08034 Barcelona, Spain

**Keywords:** Macromolecules and clusters, Ultracold gases

## Abstract

A halo is an intrinsically quantum object defined as a bound state of a spatial size which extends deeply into the classically forbidden region. Previously, halos have been observed in bound states of two and less frequently of three atoms. Here, we propose a realization of halo states containing as many as six atoms. We report the binding energies, pair correlation functions, spatial distributions, and sizes of few-body clusters composed by bosonic dipolar atoms in a bilayer geometry. We find two very distinct halo structures, for large interlayer separation the halo structure is roughly symmetric and we discover an unusual highly anisotropic shape of halo states close to the unbinding threshold. Our results open avenues of using ultracold gases for the experimental realization of halos composed by atoms with dipolar interactions and containing as many as six atoms.

## Introduction

One of the most remarkable aspects of ultracold quantum gases is their versatility, which permits to bring ideas from other areas of physics and implement them in a clean and highly controllable manner. Some of the examples of fruitful interdisciplinary borrowings include Efimov states, originally introduced in nuclear physics and observed in alkali atoms^1–3^, lattices created with counter-propagating laser beams^4,5^ as models for crystals in condensed matter physics, and Bardeen–Cooper Schrieffer (BCS) pairing theory first introduced to explain superconductivity and later used to describe two-component Fermi gases^6,7^. In the present work, we exploit the tunability of ultracold gases to demonstrate the existence of halo states composed by a large number of atoms with dipolar interactions. Originating in nuclear physics^8–10^, halo dimer states have been studied and experimentally observed in ultracold gases^11^.

A halo is an intrinsically quantum object and it is defined as a bound state with a wave function that extends deeply into the classically forbidden region^12,13^. These states are characterized by two simultaneous features: a large spatial size and a binding energy which is much smaller than the typical energy of the interaction. One of the most dramatic examples of a halo system, experimentally known, is the Helium dimer ($^4\mathrm {He}_2$)^14^, which is about ten times more extended than the size of a typical diatomic molecule^11^.

While most of the theoretical and experimental studies of halos have been carried in three dimensions (3D)^15–24^, there is an increasing interest in halos in two dimensions (2D)^12,25,26^. In fact, two dimensions are especially interesting as halos in 2D have different properties^12^ of the 3D ones. A crucial difference between 3D and 2D geometries is that lower dimensionality dramatically enhances the possibility of forming bound states. If the integral of the interaction potential $V(\rho )$ over all the space is finite and negative, $V_{k=0} = \int V({\varvec{\rho }})d{\varvec{\rho }}<0$, this is always sufficient to create a two-body bound state in 2D but not necessarily in 3D. Furthermore, the energy of the bound-state is exponentially small in 2D and it can be expressed as $E = -\hbar ^2/(2ma^2) \exp [-\hbar ^2|V_{k=0}|/(2\pi m)]$^27^, where *a* is the typical size of the bound state. An intriguing possibility arises when such integral is exactly equal to zero, $V_{k=0} = 0$, as a priori it is not clear if a bound state exists. This situation exactly happens in a dipolar bilayer in which atoms or molecules are confined to two layers separated by a distance *h* and the dipolar moments *d* are aligned perpendicular to the plane of motion by an external field. The interaction between atoms of different layers is given by $V(\rho ) = d^2(\rho ^2-2h^2)/(\rho ^2+h^2)^{5/2}$, where $\rho$ is the in-plane distance. The vanishing Born integral has first lead to conclusions that the two-body bound state disappears when the distance between the layers is large^28^ although later it was concluded that the bound state exists for any separation^29–34^, consistently with Ref.^35^. A peculiarity of this system is that the bound state is extremely weakly bound in $h\rightarrow \infty$ limit. That is, a potential with depth $V(\rho =0) = -d^2/h^3$ and width *h* would be expected to have binding energy equal to $E = -\hbar ^2/(2ma^2)\exp (-\text {const}\cdot r_0/h)$ where $r_0 = md^2/\hbar ^2$ is the characteristic distance associated with the dipolar interaction and *m* is the particle mass. Instead, the correct binding energy, $E = - 4\hbar ^2/mh^2\exp (-8r_0^2/h^2 + O(r_0/h))$^31,32^ is much smaller as it has $h^{-2}$ in the exponent and not the usual $h^{-1}$. This suggests that the bilayer configuration is very promising for the creation of a two-body halo state. Moreover, the peculiarity of the bilayer problem has resulted in the controversial claim that the three- and four-body^36^ bound states never exist in this system, and only very recently it has been predicted that actually, they are formed^37^.

In the present work, we analyze the ground-state properties of few-body bound states of dipolar bosons within a two-dimensional bilayer setup, as candidates for halo states. In particular, we study the ground-state of up to six particles occupying the A and B layers, with A and B denoting particles in different planes. In order to find the exact system properties, we rely on the diffusion Monte Carlo (DMC) method^38^ with pure estimators^39^, which has been used previously to get an accurate description of quantum halo states in Helium dimers^17^, trimers and tetramers^18,19^. In addition, we report relevant structure properties of the clusters, such as the spatial density distributions and the pair distribution functions for characteristic interlayer separations.

## Hamiltonian

We consider two-dimensional systems consisting from two to six dipolar bosons of mass *m* and dipole moment *d* confined to a bilayer setup. All the dipole moments are oriented perpendicularly to the layers making the system always stable. In this configuration the angular dependence of the dipolar interaction vanishes. In our model, we suppose that the confinement to each plane is so tight that there is no interlayer tunneling and excitations into the excited levels of the tight confinement are suppressed. The Hamiltonian of this system is

1 $$\begin{aligned} H=-\frac{\hbar ^2}{2m}\sum _{i=1}^{N_{\text{ A }}}\nabla ^2_i-\frac{\hbar ^2}{2m} \sum _{\alpha =1}^{N_{\text{ B }}}\nabla _\alpha ^2 +\sum _{i<j}\frac{d^2}{\rho ^3_{ij}}+\sum _{\alpha <\beta }\frac{d^2}{\rho ^3_{\alpha \beta }}+\sum _{i\alpha }\frac{d^2(\rho _{i\alpha }^2-2h^2)}{(\rho _{i\alpha }^2+h^2)^{5/2}}, \end{aligned}$$

where *h* is the distance between the layers. The terms in the first row of the Hamiltonian (1) are the kinetic energy of $N_{\text{ A }}$ dipoles in the bottom layer and $N_{\text{ B }}$ dipoles in the top layer; the first two terms in the second row correspond to the intralayer dipolar interactions of $N_{\text{ A }}$ and $N_{\text{ B }}$ bosons; and the last one accounts for the interlayer interactions. The in-plane distance between two bosons belonging to the same layer is denoted by $\rho _{ij(\alpha \beta )}=|{\varvec{\rho }}_{i(\alpha )}-{\varvec{\rho }}_{j(\beta )}|$, and belonging to different layers by $\rho _{i\alpha }=|{\varvec{\rho }}_{i}-{\varvec{\rho }}_{\alpha }|$, where $\rho _i$ is the in-plane position. We use the characteristic dipolar length $r_0=md^2/\hbar ^2$ and energy $E_0=\hbar ^2/(mr_0^2)$ as units of length and energy, respectively. We use $\rho$ for 2D in-plane distances and *r* for 3D distances.

Dipoles in the same layer are repulsive, with an interaction decaying as $1/\rho ^3$. However, for dipoles in different layers the interaction is attractive for small in-plane distance $\rho$ and repulsive for larger $\rho$. In other terms, a dipole in the bottom layer induces attractive and repulsive zones for a dipole in the top layer. Importantly, the area of the attractive cone increases with the distance between layers *h*, making the formation of few-body bound states more efficient.

## Structure of the bound states

We first analyze the structure of few-body clusters, composed of two, three or four particles, as a function of the interlayer separation *h*. To this end, we calculate the pair distribution function $g_{\sigma \sigma ^{'}}(r)$, which is proportional to the probability of finding two particles at a relative distance *r*. In the case of the ABB trimers and AABB tetramers, we also determine the ground-state density distributions for different values of the interlayer separation.

### AB dimer

**Figure 1:**
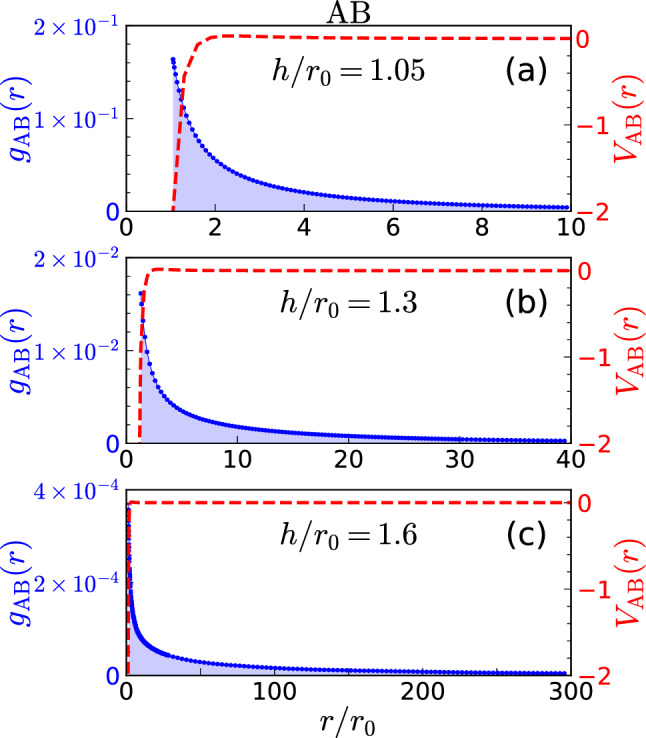
Interlayer pair distributions $g_{{\text{ A }}{\text{ B }}}(r)$ (left-axis, blue curves) and dipolar potentials $V_{{\text{ A }}{\text{ B }}}(r)$ (right-axis, red curves) for AB and for three values of the interlayer distance $h/r_0=1.05$, 1.3 and 1.6. Notice the different scales in the *r* axis.

The AB dimer is strongly bound for $h\lesssim r_0$ and its energy decays exponentially in the limit of large interlayer separation^32^. In order to understand how the cluster size changes with $h/r_0$, we show in Fig. 1 the interlayer pair distributions $g_{{\text{ A }}{\text{ B }}}(r)$ (left-axis, blue curves) for three values of $h/r_0$. The strong-correlation peak of $g_{{\text{ A }}{\text{ B }}}$ at $h/r_0$ is due to the interlayer attraction $V_{{\text{ A }}{\text{ B }}}(r)$ at short distances, also shown in the right-axis of the figure (red curves). For the cases shown in Fig. 1 we notice that $g_{{\text{ A }}{\text{ B }}}$ are very wide in comparison to the interlayer distance $h/r_0$ reflecting the exponential decay of the bound state. The tail at large distances becomes longer as the interlayer distance increases.

### ABB trimer

**Figure 2:**
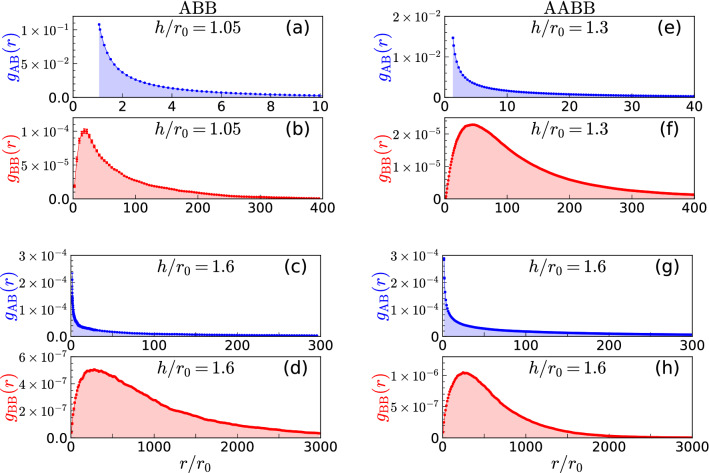
Interlayer and intralayer pair distributions, $g_{{\text{ A }}{\text{ B }}}(r)$ and $g_{{\text{ B }}{\text{ B }}}(r)$, for ABB (**a**, **b**, **c**, **d**) and AABB (**e**, **f**, **g**, **h**) clusters, and for different values of the interlayer distance $h/r_0$.

The ABB trimer is bound for large enough separation between the layers $h/r_0>0.8$ while, for smaller separations, it breaks into a dimer and an isolated atom^37^. The trimer binding energy is vanishingly small for $h\approx h_c$, with $h_c\simeq 0.8 r_0$, and it becomes larger as *h* is increased, reaching its maximum absolute value at $h/r_0\approx 1.05$. Then, it vanishes again in the limit of $h\rightarrow \infty$^37^. We report the intralayer and interlayer pair distributions, $g_{{\text{ B }}{\text{ B }}}(r)$ and $g_{{\text{ A }}{\text{ B }}}(r)$ respectively, in Fig. 2 for strongly- (a, b) and weakly-bound (c, d) trimers. We observe that the $g_{{\text{ A }}{\text{ B }}}$ distributions are very wide in comparison to *h*, similarly to what has been observed in Fig. 1 for dimers. The same-layer distribution $g_{{\text{ B }}{\text{ B }}}$ vanishes when $r/r_0 \rightarrow 0$ as a consequence of the strongly repulsive dipolar intralayer potential at short distances. As *r* increases, $g_{{\text{ B }}{\text{ B }}}$ exhibits a maximum, next it monotonically decreases with $r/r_0$. For a weakly-bound trimer ($h/r_0=1.6$), both $g_{{\text{ A }}{\text{ B }}}$ and $g_{{\text{ B }}{\text{ B }}}$ produce long tails at large distances.

**Figure 3:**
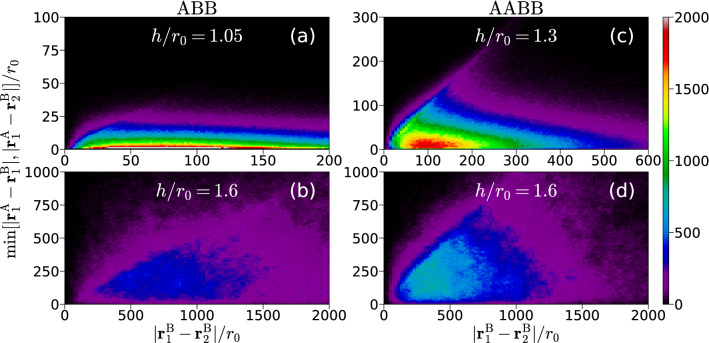
Spatial structure of the ground-state for ABB trimer (**a**, **b**) and AABB tetramer (**c**, **d**) for different values of the interlayer distance. The distance between two dipoles in the same layer is plotted in the horizontal axis and the minimum distance between dipoles in different layers is shown in the vertical axis. Plots created using Matplotlib (version 3.3.3)^40^.

The trimer is weakly bound close to the threshold, $h\rightarrow h_c$, and for large interlayer separation, $h\rightarrow \infty$, but its internal structure in those two limits is significantly different. This can be seen in Fig. 3a, b, where we plot the trimer ground-state spatial distribution for $h/r_0=1.05$ and 1.6. The spatial distribution is shown as a function of the distance between two dipoles in the same layer $|\mathbf{r}_1^{{\text{ B }}}-\mathbf{r}_2^{{\text{ B }}}|$ (horizontal axis) and the minimal distance between dipoles in different layers $\mathrm {min}\{|\mathbf{r}_1^{{\text{ A }}}-\mathbf{r}_1^{{\text{ B }}}|, |\mathbf{r}_1^{{\text{ A }}}-\mathbf{r}_2^{{\text{ B }}}|\}$ (vertical axis). For large separation between layers, shown in Fig. 3b for $h/r_0=1.6$, the distances between AB and BB atoms are all of the same order, revealing an approximately symmetric structure. However, by decreasing the distance between layers the particle distribution becomes significantly asymmetric. For $h/r_0=1.05$ (Fig. 3a), we observe that the trimer spatial distribution is elongated: two dipoles in different layers are close to each other while the third one is far away. Regardless of the interlayer separation, the pair ${\text{ A }}{\text{ B }}$ is, on average, closer than the ${\text{ B }}{\text{ B }}$ pair. As the threshold value is approached, the trimer becomes more extended and breaks into a dimer and a single atom at $h/r_0\approx 0.8$.

### AABB tetramer

As we have shown in the previous Section, an ABB trimer dissolves into a dimer and an atom when $h\approx h_c$. Here, we address the structure properties of the balanced case for a tetramer, in which the number of A and B atoms is the same. The AABB tetramer is weakly bound for large values of $h/r_0$. When the distance between layers decreases, the tetramer becomes unbound at $h/r_0\approx 1.1$ and splits into two AB dimers^37^.

The pair distributions $g_{{\text{ A }}{\text{ B }}}$ and $g_{{\text{ B }}{\text{ B }}}$ for AABB are shown in Fig. 2e–h for two characteristic values of the interlayer distance $h/r_0$. We observe a behavior that is similar to that previously reported for ABB. That is, both $g_{{\text{ A }}{\text{ B }}}$ and $g_{{\text{ B }}{\text{ B }}}$ are compact for the deepest bound state ($h/r_0=1.3$ for AABB) and become diffuse, showing long tails at large distances, when it turns to a weakly-bound state ($h/r_0=1.6$).

The ground-state spatial distributions for the symmetric tetramer are shown in Fig. 3c, d. We observe that for large separation *h*, i.e., when the tetramer is weakly bound, it has large spatial extension and the distances between AA and AB pairs are of the same order. As the interlayer separation is progressively decreased, the tetramer size decreases and its structure becomes anisotropic. In this case, the distance between dipoles in the same layer is several times larger than the distance between dipoles in different layers. When the tetramer approaches the threshold for unbinding the cluster becomes even more elongated and it breaks into two AB dimers at $h/r_0\approx 1.1$.

## Quantum halo characteristics

A halo is a quantum bound state in which particles have a high probability to be found in the classically forbidden region, outside the range of the interaction potential. The key characteristics of a halo are its extended size and binding energies much smaller than the typical energy of the interaction. In order to classify a system as a halo state, one typically introduces two scaling parameters with which the size and the energy are compared. The first parameter is the scaling length *R*. For two-body systems one commonly chooses *R* as the outer classical turning point. The second parameter is the scaling energy $\mu BR^2/\hbar ^2$, where $\mu$ is the reduced mass and *B* is the absolute value of the ground-state energy of the cluster. The size of a cluster is usually quantified through its mean-square radius $\langle \rho ^2 \rangle$, where $\rho$ is the interparticle distance. A two-body quantum halo is then defined by the condition $\langle \rho ^2 \rangle /R^2>2$, which means that the system has a probability to be in the classically forbidden region larger than 50$\%$^12^.

**Figure 4:**
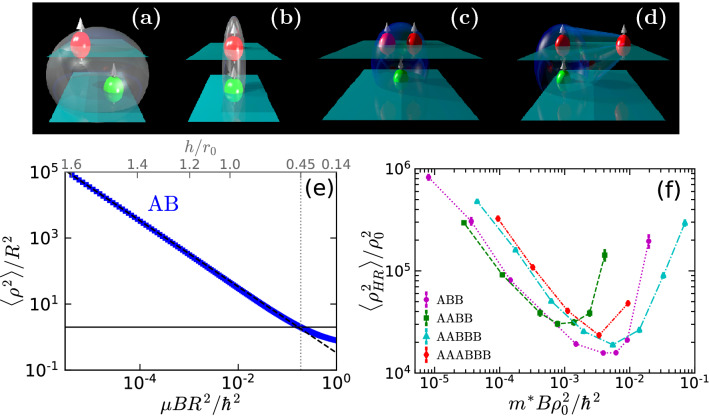
Top panel: Schematic representation of the AB and ABB states in two limits: (**a**) AB is a halo state; (**b**) AB is not a halo state; (**c**) ABB $h\rightarrow \infty$; (**d**) ABB $h\rightarrow h_c$. Figures created using POV-Ray (version 3.7)^41^. Bottom panel: (**e**) Size $\langle \rho ^2\rangle /R^2$ vs ground-state energy $\mu BR^2/\hbar ^2$ scaling plot for two-body halos. The horizontal line is the quantum halo limit and the dashed one is $\langle \rho ^2\rangle /R^2=\hbar ^2/(3\mu BR^2)$, which is a zero-range approximation for two-body halos in two-dimensions^12^. (**f**) Size $\langle \rho _{HR}^2\rangle /\rho _0^2$ vs ground-state energy $m^*B\rho _0^2/\hbar ^2$ scaling plot for three- up to six-body halos.

The dipolar interaction in the bilayer geometry has vanishing Born integral and thus, the AB dimer can show an enhancement of its halo properties. In Fig. 4e, we show the scaling plot for the dipolar dimers, corresponding to interlayer distance from $h/r_0=0.14$ to 1.6, as indicated on the upper axis. All dimers which lie above the halo limit $\langle \rho ^2\rangle /R^2=2$ (horizontal line in Fig. 4e) are halo states and follow a universal scaling law $\langle \rho ^2\rangle /R^2=\hbar ^2/(3\mu BR^2)$, shown with a dashed line in the figure^12^. This is exactly the case for all dimers with interlayer separations $h/r_0 > 0.45$. This threshold value is close to the characteristic value, $h/r_0=0.5$, for which the dimer binding energy is approximately equal to the typical energy of the dipolar interaction $E_{{\text{ A }}{\text{ B }}} \approx \hbar ^2/(mr_0^2)$.

While AB dimers exist for any interlayer separation, ABB trimers and AABB tetramers are self-bound for large *h* values, where AB dimers are in fact halo states. Thus, it can be anticipated that these few-body bound states are also halos. To verify that, the sizes of three- and four-body systems should be compared to the mean-square hyperradius^12^,

2 $$\begin{aligned} \rho _{HR}=\sqrt{\frac{1}{m^*M}\sum _{i<k}m_im_k({\varvec{\rho }}_i-{ \varvec{\rho }}_k)^2}, \end{aligned}$$

where $m^*$ is an arbitrary mass unit, *M* is the total mass of the system, and $m_i$ is the mass of particle *i*. In analogy to the hyperradius Eq. (2), Jensen et al.^12^ defined the scaling size parameter $\rho _0$ as

3 $$\begin{aligned} \rho _0=\sqrt{\frac{1}{m^*M}\sum _{i<k}m_im_kR_{ik}^2}, \end{aligned}$$

with $R_{ik}$ the two-body scaling length of the $i-k$ system, which is calculated as the outer classical turning point for the $i-k$ potential. Considering the problem of a dipolar dimer in the bilayer, the two-body scaling length $R_{E,2}$ is related to the interlayer interaction potential $V_{{\text{AB }}} (\rho ,h)$ and the dimer energy $E_2$ according to

4 $$\begin{aligned} V_{{\text{AB }}} (R_{E,2},h)=\frac{d^2(\rho ^2-2h^2)}{(\rho ^2+h^2)^{5/2}}\Bigr |_{\rho =R_{E,2}} = E_2. \end{aligned}$$

For a given value of the interlayer distance *h* the classical turning points are given by the roots of Eq. (4) which is a polynomial of tenth degree in $R_{E,2}$. The exact value of the dimer energy $E_2$ depends on *h* and can be obtained by solving numerically the Schrödinger equation with the potential $V_{{\text{AB }}} (\rho ,h)$ or using the DMC method. In Table 1, we report the classical turning points $R_{E,2}$ corresponding to interlayer distance from $h/r_0=0.4$ to 1.6. On the other hand, for repulsive potentials we choose $R_{ik}$ equal to zero. The condition for three- and four-body quantum halos is then $\langle \rho ^2_{HR} \rangle / \rho _0^2>2$^12^.

**Table 1:** Interlayer distances $h/r_0$ and classical turning points $R_{E,2}/r_0$ for a two-body system in a dipolar bilayer.

$h/r_0$	$R_{E,2}/r_0$
0.4	0.3874
0.6	0.6924
0.8	1.0364
1.0	1.3776
1.2	1.6890
1.4	1.9789
1.6	2.2627

**Table 2:** Classical turning points $R_{E,2}$, $R_{B,N}$ and $R_{pot,N}$, defined by Eqs. (4–6) in *N*-body system in a dipolar bilayer with $h/r_0=1.2$.

*N*	$R_{E,2}/r_0$	$R_{E,N}/r_0$	$R_{pot,N}/r_0$
2	1.6889	1.6889	1.6055
3	1.6889	1.6941	1.6454
4	1.6889	1.6943	1.6431
5	1.6889	1.6952	1.6625
6	1.6889	1.6954	1.6634

While there is a general agreement on the definition of the classical turning point $R_{E,2}$ in the two-body problem, Eq. (4), in the few-particle case there is not a single way to determine the scaling length $R_{ik}$. It has been suggested^24^ to consider $R_{ik}$ as the distance at which the attractive interaction Eq. (4) equals a characteristic energy of the system. One possibility is to obtain the classical turning point from the relation,

5 $$\begin{aligned} V_{{\text{AB }}} (R_{pot,N},h) = \langle V_{{\text{AB }}} \rangle , \end{aligned}$$

where the brackets $\langle \cdots \rangle$ denote averaging of the two-body interaction potential over the many-body bound state. Another possibility^24^, denoted by $R_{E,N}$, is based on comparing the two-body potential to the bound-state energy $B_{N}$ divided by the number of pairs $N(N-1)/2$,

6 $$\begin{aligned} V_{{\text{AB }}} (R_{E,N},h) = \frac{2}{N(N-1)}B_{N}. \end{aligned}$$

Even while for large values of *h*, the average potential energy is similar in absolute value to the average kinetic energy and is large compared to the bound-state energy $B_N$, energies in Eqs. (5, 6) are so tiny that in both cases $R_{pot,N}$ and $R_{E,N}$ are close to the position where $V_{{\text{AB }}} (R_{cl})=0$. In Table 2, we report the values of $R_{E,2}$, $R_{E,N}$, and $R_{pot,N}$ for $h/r_0=1.2$ and for $N=2,\ldots ,6$. One notices that in the bilayer system the three different definitions, Eqs. (4–6), result in very similar values. Therefore, choosing one of these radii over the other two to calculate $\rho _0$ is a minor effect. In the following we will use $R_{E,2}$ to calculate the scaling size parameter $\rho _0$.

The dependence of the scaled size on the scaled energy for ABB and AABB are shown in Fig. 4f. We find a non-monotonic behavior, in clear contrast with the dependence observed in the dimer case (see Fig. 4e). That is, the cluster size decreases with increasing energy and reaches a minimum and then it starts to grow again. The minima correspond to the deepest bound states^37^. This resurgence appears as the clusters approach the thresholds, where trimers eventually break into a dimer and an atom, and tetramers into two dimers. We want to emphasize that all the trimers and tetramers analyzed in Fig. 4 are halo states, although they are organized in significantly different spatial structures. On the left side of the minima, the clusters are almost radially symmetric and all the interparticle distances are of the same order. However, at the minima and on the right side of the minima the cluster structures are elongated and highly asymmetric. The AABBB pentamer and AAABBB hexamer are self-bound and are manifestly halo states. Their mean square size has a similar behavior to the one observed before for the trimer and tetramer, that is a minimum corresponding to the larger binding energy which separates a regime of nearly symmetric particle distribution from another one, more elongated, and thus asymmetric. It is important to notice that the existence of halo tetramers, pentamers and hexamers is very unusual as it contradicts the usual tendency of self-bound clusters to shrink and lose the halo character as the number of particles is increased. As we demonstrate in the present work, the bilayer geometry is very promising for creation of halo states with up to six particles.

Experimental signatures of halo and Efimov states come from the measurement of three- and four-body recombination loss rate in ultracold gases^2,42,43^ or by using techniques such as matter-wave diffraction combined with laser-induced Coulomb explosion imaging^14,44^. Using the latter techniques the halo states of helium dimer^14^ and trimer^44^ were detected by measuring the binding energies and pair distances distributions. A possible experimental implementation for observing the predicted halo states can be realized by using bosonic dipolar molecules produced with mixtures of $^{87}$ $\hbox {Rb}^{133}$Cs^45,46^ and $^{23}$ $\hbox {Na}^{87}$Rb^47,48^ characterized by dipolar lengths $r_0\sim 5\times 10^{-6}$m and $2\times 10^{-5}$m, respectively. Magnetic dipolar $^{164}$ $\hbox {Dy}_2$^49^ and $^{168}$ $\hbox {Er}^{164}$Dy^50^ ($r_0\sim 2\times 10^{-7}$m and $r_0\sim 1\times 10^{-7}$m, respectively) molecules can also be used. The interlayer distance, half of the laser wavelength $\lambda$, has typical values of $h\approx (2-5)\times 10^{-7}$m, and typical lengths of the transverse confinement $a_\perp =(\lambda /2\pi )s^{-1/4}$ are $a_\perp \approx (3-8)\times 10^{-8}$m, where $s\sim 16$ is the potential depth of the transverse confinement in units of the recoil energy.

A possible issue of concern may be the validity of our findings in an experimental realization, where the bilayer has a quasi-two-dimensional geometry and not strictly two-dimensional one as in our model. To this aim, we have compared the dimer energy of our model with the dimer energy of a quasi 2D model. For typical experimental parameters, we have found that the change in the dimer energy is at most 20$\%$. Therefore, it can be concluded that the effects of considering a quasi-two dimensional model do not change our main findings.

## Conclusions

We used the diffusion Monte Carlo method to study the ground-state properties of few-body bound states of dipolar bosons in a two-dimensional bilayer setup. We have studied clusters composed by up to six particles, for different values of the interlayer distance, as candidates for quantum halo states.

In the case of dimers, we find that for values of the interlayer separation larger than $h/r_0 = 0.45$ the clusters are halo states and they follow a universal scaling law. In the cases of trimers up to hexamers, we find two very different halo structures. For large values of the interlayer separation the halo structures are almost radially symmetric and the distances between dipoles are all of the same scale. In contrast, in the vicinity of the threshold for unbinding, the clusters are elongated and highly anisotropic. To our knowledge halo states have been experimentally observed for up to four particles^2,14,42–44^. The addition of particles to a two or three body halo states typically makes them shrink towards a more compact liquid structure. Importantly, our results prove the existence of stable halo states composed by atoms with dipolar interactions and containing up to six particles. As commented before, there are reasons to believe that our system is experimentally viable with current technology as compared to other theoretical predictions, where zero- and finite-range interactions are used^23,24^. We conclude that the bilayer geometry is advantageous for the observation of halo states in future experiments. We hope that these results will stimulate experimental activity in this setup, composed by atoms with dominant dipolar interaction, to bring evidence of these quantum halo states.

In outlook, our results can stimulate further theoretical and experimental research of halo states in ultracold gases. It could be interesting to understand how the possibility of tunneling between the layers affects the stability of the halo states. An interesting new path of research could be to study halo states in a bilayer system of fermionic dipoles^51,52^.

## Method

To investigate the structural properties of the dipolar clusters, we use a second-order DMC method^38^ with pure estimators^39^. This method allows for an exact estimation of the ground-state energy, as well as other properties, within controllable statistical errors. The DMC stochastically solves the imaginary-time Schrödinger equation,

7 $$\begin{aligned} -\frac{\partial \Psi (\varvec{\rho },\tau )}{\partial \tau }=(H-E_s) \Psi (\varvec{\rho },\tau ), \end{aligned}$$

where $\tau =it/\hbar$ is a imaginary time, $E_s$ is an energy shift and the walker $\varvec{\rho }=(\varvec{\rho }_1,\dots ,\varvec{\rho }_N)$ is a vector containing positions of *N* particles. Importance sampling is used to reduce the statistical noise of the calculation, which consists on rewritten the Schrödinger equation, Eq. (7), for the mixed distribution $\Phi (\varvec{\rho },\tau )=\Psi (\varvec{\rho },\tau )\psi (\varvec{\rho })$. We use a trial wave function $\psi$ of the form

8 $$\begin{aligned} \psi (\varvec{\rho }_1,\dots ,\varvec{\rho }_N)=\prod _{i<j}^{N_{\text{ A }}}f_{{\text{ A }}{\text{ A }}} (\rho _{ij})\prod _{\alpha <\beta }^{N_{\text{ B }}}f_{{\text{ B }}{\text{ B }}}(\rho _{\alpha \beta }) \left[ \prod _{i=1}^{N_{\text{ A }}}\sum _{\alpha =1}^{N_{\text{ B }}}f_{{\text{ A }}{\text{ B }}}(\rho _{i\alpha })+ \prod _{\alpha =1}^{N_{\text{ B }}}\sum _{i=1}^{N_{\text{ A }}}f_{{\text{ A }}{\text{ B }}}(\rho _{i\alpha })\right] , \end{aligned}$$

which is suitable for describing systems with short-range correlations and as well as long-range asymptotic behavior.

The trial wave function for intraspecies correlations is built from the zero-energy two-body scattering solution

9 $$\begin{aligned} f_{{\text{ A }}{\text{ A }}}(\rho )=f_{{\text{ B }}{\text{ B }}}(\rho )=C_0K_0(2\sqrt{r_0/\rho }), \end{aligned}$$

$K_0(\rho )$ being the modified Bessel function and $C_0$ a constant. The interspecies interactions are described by the dimer wave function $f_{{\text{ A }}{\text{ B }}}(\rho )$ up to $R_0$. From the variational distance $R_0$ on we took the free scattering solution $f_{{\text{ A }}{\text{ B }}}(\rho )=CK_0(\sqrt{-mE_{{\text{ A }}{\text{ B }}}}\rho /\hbar )$. We impose continuity of the logarithmic derivative at the matching point $R_0$, yielding the following equality

10 $$\begin{aligned} \frac{f^{'}_{{\text{ A }}{\text{ B }}}(R_0)}{f_{{\text{ A }}{\text{ B }}}(R_0)}=-\frac{\sqrt{-mE_{{\text{ A }}{\text{ B }}}}}{\hbar } \frac{K_1(\sqrt{-mE_{{\text{ A }}{\text{ B }}}}R_0/\hbar )}{K_0(\sqrt{-mE_{{\text{ A }}{\text{ B }}}}R_0/\hbar )}. \end{aligned}$$

In a DMC calculation, the expectation value of an observable ${\hat{O}}$ is obtained for long enough imaginary time propagation

11 $$\begin{aligned} \langle {\hat{O}}\rangle _{mix}=\frac{\langle \psi |{\hat{O}}|\Psi _0\rangle }{\langle \psi |\Psi _0\rangle }=\lim _{\tau \rightarrow \infty }\frac{\int d\varvec{\rho }\psi (\varvec{\rho }) {\hat{O}} \Psi (\varvec{\rho },\tau ) }{\int d\varvec{\rho }\psi (\varvec{\rho }) \Psi (\varvec{\rho },\tau )}, \end{aligned}$$

with $\Psi _0$ the ground-state wave function. The last equation is known as the *mixed estimator*. Equation (11) gives the exact expectation value for the Hamiltonian and for observables that commute with it. In the case of operators that do not commute with ${\hat{H}}$, the result obtained from Eq. (11) can be biased by $\psi$. In this case, it is possible to obtain exact expectation values using the pure estimators technique^39^. In the present study, pure estimators are used for the calculation of the pair distribution functions, the spatial distribution functions, and the size of the clusters.
